# Application of Microfluidics in Experimental Ecology: The Importance of Being Spatial

**DOI:** 10.3389/fmicb.2018.00496

**Published:** 2018-03-20

**Authors:** Krisztina Nagy, Ágnes Ábrahám, Juan E. Keymer, Péter Galajda

**Affiliations:** ^1^Biological Research Centre, Institute of Biophysics, Hungarian Academy of Sciences, Szeged, Hungary; ^2^Doctoral School of Multidisciplinary Medical Science, University of Szeged, Szeged, Hungary; ^3^School of Biological Sciences and School of Physics, Pontifical Catholic University of Chile, Santiago, Chile

**Keywords:** microbial ecology, spatial ecology, microfluidics, communities, microbial interactions

## Abstract

Microfluidics is an emerging technology that is used more and more in biology experiments. Its capabilities of creating precisely controlled conditions in cellular dimensions make it ideal to explore cell-cell and cell-environment interactions. Thus, a wide spectrum of problems in microbial ecology can be studied using engineered microbial habitats. Moreover, artificial microfluidic ecosystems can serve as model systems to test ecology theories and principles that apply on a higher level in the hierarchy of biological organization. In this mini review we aim to demonstrate the versatility of microfluidics and the diversity of its applications that help the advance of microbiology, and in more general, experimental ecology.

## Introduction

On all hierarchical scales in biology, from cells to the biosphere, organisms live in close interaction with each other and their environment. Ecology focuses on these interactions and their role in the formation and maintenance of complex biological systems known as ecosystems. Early ecology was mostly a quantitative and descriptive discipline, and research was largely based on observations of natural ecosystems. Nevertheless, important theoretical progress was made. Malthus was among the first to highlight the role ecosystem services play in regulating population growth (Malthus, 1798). Early demographers, like Verhulst created mathematical models to account for the density dependence of population growth (Verhulst, 1838). In those days, the predictive power of theories and models were tested by observing natural patterns (Paine, 1994). The study conducted by George Sinclair at Woburn Abbey, Bedfordshire, England is considered to be the first ecology experiment. He grew various plant communities in 242 experimental plots with various types of soil, and, among other things, he concluded that more diverse plant communities were more productive (Sinclair, 1824, 1826). Darwin referred to this work in the “On the Origin of Species” (Darwin, 1985; Hector and Hooper, 2002).

Following this early descriptive phase, ecological experiments started to gain more importance, especially in studying marine intertidal ecosystems. Conducting experiments in ecology, however, is often difficult, since reproducing the complexity of an ecological system in a laboratory environment is challenging. Thus, most ecological experiments rely on creating disturbances in natural systems (Paine, 1994). A classical set of examples were conducted in intertidal ecosystems to study the effect of predator removal (Paine, 1966), and competition between barnacle species (Connell, 1961a,b). In terrestrial ecosystems, for example, Damschen and co-workers used patches cut into forests and followed their colonization by plants in order to study the effects habitat fragmentation and ecological corridors have upon patterns of species diversity (Damschen et al., 2006, 2008). In such field experiments, the scale and complexity of the system often make it very difficult to conduct adequate control experiments and do replications (Hurlbert, 1984). Moreover, in many cases the relevant timescales (life cycle or generation times) make it infeasible to conduct sufficiently long experiments. This is demonstrated by the Park Grass Experiment, one of the longest scientific experiment, which investigates the impact of fertilization on hay yields since 1856 (Silverton et al., 2006).

Considering these difficulties, the ideal experiment in ecology would involve the free design of habitat landscapes and the creation of artificial ecosystems with sufficient complexity and control over the environmental conditions. In this mini review we collect several examples which demonstrate that combining microfluidic techniques with microbiology can bring us a step closer to this goal. In particular, we focus on experiments where the spatial aspects of the ecological processes are emphasized, since in the last few decades “the importance of being spatial” has been acknowledged widely, both in ecological theory and experiments (Legendre and Fortin, 1989; Durrett and Levin, 1994; Collinge, 2001; Green and Bohannan, 2006; Rietkerk and van de Koppel, 2008; Gonzalez et al., 2012).

## Microfluidics

Microfabrication and microfluidics (Whitesides, 2006; Qin et al., 2010; Leester-Schädel et al., 2016) offer ways to create engineered structures and manipulate fluids in microscopic dimensions. In the last few decades these techniques proved to be useful in a vast spectrum of medical, biological and chemical research (Liu and Zhu, 2005; Melin and Quake, 2007; Weibel et al., 2007; Hol and Dekker, 2014; Sackmann et al., 2014; Castillo-León, 2015; Samiei et al., 2016; Wu and Dekker, 2016; Xiao-Ying, 2016; Shang et al., 2017). Microfluidic chips consisting of channels, chambers and other structures (porous walls, slits, etc.,) may be used as engineered habitats for microbes to create artificial microscopic ecosystems (Rusconi et al., 2014). The geometry of these structures can be controlled with subcellular precision, therefore, habitats with different physical-chemical characteristics may be realized at the scale of an individual cell. This is useful, for example, in creating habitat structures of various degrees of fragmentation or connectivity, which require substantial effort in macroscopic field experiments (Damschen et al., 2006; Haddad et al., 2015). Such level of geometric control, together with the capability of liquid manipulation, makes it possible to precisely regulate the chemical environment in which microbial cells live within these devices. Additionally, the laminar nature of liquid flow on the microscale enables to accurately calculate the flow and diffusion conditions as well as optimize the design of the device based on these calculations (Squires and Quake, 2005). These capabilities of the microfluidic technology permit accurate regulation of various ecologically relevant environmental factors in experiments, such as resource supply (nutrient, oxygen, space, etc.) or interactions between organisms (e.g., by washing off compounds relevant in biochemical interactions between cells). With all the advantages of the micro-scale (high-throughput, controlled lab experiments vs. field experiments), microfluidics is restricted for use with microscopic organisms. Various microscopy imaging methods and digital image processing techniques may be used to observe and analyze microbes in microfluidic devices (Van Teeffelen et al., 2012; Obara et al., 2013; Sadanandan et al., 2015). Regardless of size restrictions, artificial microfluidic microbial ecosystems have a scientific impact that goes beyond microbial ecology and thus can serve as model systems to test and validate general or multiscale ecological theories and concepts.

## Density dependence and the dynamics of single populations

A central issue in population dynamics is the role that population density plays in determining demographic patterns. Demography is one of the largest and oldest branches of mathematical biology and it is the basis of various other sub-fields in ecology. The important questions are the following. How and why densities of organisms change in time and space? What are the basic principles and mechanisms that govern these changes? How do various environmental factors affect the dynamics? The basic exponential law of uncontrolled growth (Malthus, 1798), as well as the simplest controlled growth model described by the logistic equation (Verhulst, 1838) can be applied well to microbial cultures. As Jacques Monod wrote, “The study of the growth of bacterial cultures does not constitute a specialized subject or a branch of research: it is the basic method of microbiology” (Monod, 1949). Measuring growth curves is still the primary experimental technique to study the effect of various factors (nutrients, drugs, etc.) on the cell. From an ecological point of view, with these measurements we determine the population dynamics of the culture in various conditions. Thus, it is natural that traditional culturing methods, such as batch cultures and chemostats are often used in microbial ecology experiments (Jannasch, 1974; Veldkamp, 1977; Bohannan and Lenski, 1997; Fussmann et al., 2000; Carrero-Colón et al., 2006).

Microfluidic chemostats and micro-bioreactors make it possible to study small populations of microbes in static or controlled dynamic environments (Zanzotto et al., 2004; Hegab et al., 2013; Yang and Wang, 2016), where the small size of these devices enables running several experiments in parallel, offering high-throughput capabilities (Zanzotto et al., 2004). In these devices, bacterial populations can be continuously monitored with single-cell resolution under the microscope, so dynamic changes in the population together with morphological characteristics of individual cells can be observed. For example, Balagadde et al. used a novel microchemostat for long-term monitoring of small *E. coli* populations (10^2^–10^4^ cells) with an artificial population control mechanism achieved using a “killer gene” regulated by quorum sensing (Balagaddé et al., 2005). While in macroscale chemostats the population control was lost within 10 h, in the microchemostat the population was stably maintained and observed for hundreds of hours. In similar microfluidic reactors one can monitor colony formation from single cells and record the growth curves of these colonies over long periods of time (Groisman et al., 2005; Frey et al., 2015). Such devices are becoming popular for screening microbial agents. They offer the possibility to study the effects exogenously added signaling molecules have on the growth dynamics of microbial populations (Groisman et al., 2005).

Single-cell level observations and tracking spatial localization of cells enable to trace cell lineages during experiments, as demonstrated by Wakamoto and co-workers, who studied the dynamics of persistence of *Mycobacteria* in the presence of isoniazid drug (Wakamoto et al., 2013). They measured the killing kinetics at single-cell level by culturing bacteria in microfluidic devices and using time-lapse microscopy.

Droplet microfluidics offers a unique solution to manipulate and monitor the continuous growth of bacterial populations in numerous parallel chemostats. Hundreds of microdroplets can be created with a precise control over the chemical composition and cell number within each droplet (Jakilea et al., 2013). Although droplet-based systems are not ideal for spatial investigations, these systems offer great advantages when the goal is simply to determine growth curves of microbes as a function of different drugs or to study the effect of fluctuating/changing but otherwise well-mixed chemical environments on microbial populations. It also proved to be effective in the rapid identification of strains resistant to various treatments (Keays et al., 2016).

The above examples demonstrate that microfluidic devices can be used in place of traditional techniques in order to accomplish two things: (i) miniaturization and parallelization; (ii) simultaneous observations on the level of individuals and populations. These capabilities make it possible to gather more data with better statistics, and perform complex multi-scale analysis, which is often difficult in ecology experiments.

## Spatially structured habitats, collective migrations and metapopulations

Microfabrication technologies allow engineering environments with structured topologies that mimic the complexity of natural habitats (e.g., soil, gut), which are made of heterogeneous landscape elements creating a network of discrete locations. These confined, discrete microenvironments lead to characteristic features of the population dynamics that otherwise would be hidden in homogenous chemostat experiments or batch cultures. Populations which are spatially distributed sometimes subdivide into a multi-scale network of sub-populations, which is called metapopulation (Levins, 1969). In a fragmented landscape, these subpopulations exhibit dynamic fluctuations and oscillations as a result of the interplay between colonization and extinction processes with stochastic factors. Since the introduction of the metapopulation concept (Levin, 1970), metapopulation biology became a large field in biology (Hanski and Gilpin, 1997). Metapopulation theories are mostly based on field observations rather than controlled laboratory experiments, and therefore microfabrication techniques might be very useful to explore the importance that landscape structure and stochasticity have in determining patterns of microbial distribution and abundance.

Keymer and co-workers used a linear array of coupled microscale chambers (habitat patches) connected by corridors to culture *E. coli* bacteria (Keymer et al., 2006). After loading the cells into the device they observed the formation of a metapopulation, i.e., a collection of interacting subpopulations exhibiting dynamic patterns of spatial distribution. Such dynamic fluctuations in cell-density are assigned to migration and extinction processes operating in such an ecological system. Interestingly, van Vliet and co-workers found that the spatial segregation of populations on habitat landscapes depended on culture history (van Vliet et al., 2014). While different cultures of the same *E. coli* strain remained segregated when colonizing the same patchy landscape, co-culturing before the experiment in a batch culture led to mixed colonization. By repeating the experiments with physically separated, but chemically coupled populations, they proved the role biochemical interactions between cells play in modulating spatial competition through diffusing molecules. This highlighted the fact that interactions between organisms also contribute to the spatiotemporal population dynamics of microbes.

Park and co-workers used a microfluidic maze, i.e., a structured, but non-patchy habitat to culture bacteria (Park et al., 2003). In this case, despite the lack of patchiness, bacteria organized into complex dynamic patterns. Traveling waves of high-density *E. coli* populations were observed in the maze structures. This was not a result of colonization and extinction events, but rather due to the chemotactic response of cells to self-excreted chemoattractants in a nutrient depleted closed system. In another landscape, consisting of a central patch in an otherwise homogeneous environment, the waves nucleated population collapse of bacteria into the smallest confining structures, a phenomenon that may contribute to the survival of bacteria under stress and which underlies the collective migration patterns which sustain patch colonization.

These experiments show that microfabrication is suitable for creating complex engineered habitats and landscapes with relative ease. Structures may be designed to explore the intricate and tight interplay between habitat structure and population structure. Such level of engineering is rarely available in macroscopic ecology experiments.

## Intraspecific competition and the role of communication

Individuals of the same species may interact with each other in various ways. A basic ecological interaction, which is at the heart of the density dependence discussed earlier, is intraspecific competition. It is a widespread ecological phenomenon: individuals of the same species compete for the same resources. Direct or indirect interactions (i.e., interference or exploitation) can be observed in natural systems, and these lead to specific competitive scenarios. Various models emphasize different aspects of intraspecific competition (e.g., type or range of competition, time lags, territoriality, etc.), and microfluidics enables us to study the spatial aspects of different scenarios of intraspecific competition within microbial populations.

Nagy and co-workers used porous membranes in combination with microfluidic structures to study the interaction of physically separated, but chemically coupled *E. coli* populations (Nagy et al., 2014). They observed a dynamic pattern of interactions between neighboring populations and the resulting chemotactic spatial rearrangement of cells. Both indirect interactions (i.e., competing for the same nutrient pool), and direct interactions (through excreted factors, like indole) can play a role in the observed dynamic behavior of these coupled populations.

A special form of interaction among microbial organisms is chemically mediated, short-distance communication via secreting signaling molecules (Waters and Bassler, 2005), as well as long-distance electric communication via membrane depolarization (Liu et al., 2017). Although not an ecological phenomenon itself, communication is the basis for the transition between an ecology of independent cells into the coherent physiology of a cell-collective acting as a tissue; an issue at the core of theoretical biology. Communication has a profound effect on the behavior of individuals or populations, and it is involved in the collective or social behavior of various organisms (West et al., 2007; Zhao et al., 2017), ranging from insects (Leonhardt et al., 2016) to whales (Tyack, 1986), and humans.

A basic mechanism in bacterial communication is quorum sensing, where simultaneous excretion and detection of specific signal molecules allow a population or community to synchronize gene expression and/or behavior. By genetic manipulations of the quorum sensing mechanisms and the microfluidic control of the signal level, the role and importance of communication in the behavior of individuals or populations can be studied in detail. For example, cells or populations can be separated in space in different chambers, while the biochemical connection between them can be realized through porous structures. Park and co-workers however harvested the physical interaction of swimming cells with arrowhead-shaped structures to concentrate swimming bacteria into distant chambers while maintaining chemical connections between them (Park et al., 2012). The device was used to examine the effect of population density and spacing distance in the communication process. Similarly, micro-3D printing and scanning electrochemical microscopy can be combined to probe quorum sensing dynamics in *Pseudomonas aeruginosa* in miniature traps (Connell et al., 2014). By measuring the local concentration of the quorum sensing controlled secondary metabolite pyocyanin, the authors determined the minimal population size to be circa 500 cells, in which quorum sensing occurs. However, about 2,000 cells were needed to induce quorum sensing between cell aggregates located at 8 μm distance. It has also been shown that extreme spatial confinement of single bacterial cells results in the activation of quorum sensing pathways by the containment of self-excreted signals (Boedicker et al., 2009; Carnes et al., 2010). Luo and co-workers on the other hand created a simplified microfluidic model system to study the auto-inducer-2 based signaling in the human gastro-intestinal tract (Luo et al., 2015).

Microfluidic technologies offer an exceptionally high level of control over factors that affect intraspecific competition. Not only the spatial structure of populations and resources are controlled precisely by using engineered structures but other ecological interactions can also be tweaked by affecting diffusible signals.

## The role of space in competition, cooperation, and food web formation

Similarly to intraspecific competition, interspecific competition plays a fundamental role in determining patterns of community assembly in microbial ecosystems. Not only it affects the spatiotemporal dynamics of populations of different species, but it also has evolutionary aspects. One fundamental question is: what are the conditions and ecological factors that result in coexistence or competitive exclusion? Bacterial experiments using microfluidic techniques may contribute to answering this question. For example, microfluidic chips can be used to experimentally investigate predictions of game theory models on the competition of cooperating and cheating strategies (Lambert et al., 2014b). Hol and co-workers performed competition experiments with rpoS wild type *E. coli* bacteria (cooperators) and rpoS mutants with a growth advantage in stationary phase phenotype (cheaters) probing the role of cooperation in long-term microbial sustainability (Hol et al., 2013). They have shown that competition in mixed cultures results in remarkably different population dynamics in patchy and homogeneous environments. While cheaters outcompeted cooperators in the homogeneous case, the latter was able to sustain a stable population on patchy landscapes. It has been suggested that local segregation of the strains may have contributed to this outcome (Keymer et al., 2008).

Predator-prey interaction is another fundamental interspecific interaction which determines the structure of many ecosystems. Spatial structure and the degree of habitat patchiness can profoundly influence predator-prey dynamics. Hol and co-workers studied the influence of patchiness on bacterial predator-prey dynamics in spatially distributed ecosystems (Hol et al., 2016b). They compared the preying of *Bdellovibrio bacteriovorus* upon *E. coli* in patchy and continuous habitat landscapes. In the case of patchy habitats, a majority of *E. coli* bacteria became sessile and formed surface-associated biofilms, while in the non-fragmented environment they remained mostly planktonic. This lifestyle switch of *E. coli* probably contributed to the increased persistence of the prey population in the patchy habitat, when compared to the continuous habitat, indicating that it can significantly influence predator-prey dynamics.

Instead of defining spatial patchiness by physical confinement, one can use microfluidics to create diffusing nutrient patches for marine bacteria (Seymour et al., 2009). Similar nutrient plumes are thought to exist in marine environments. Seymour and co-workers observed a change in the swimming behavior and accumulation of bacteria in response to these plumes and found that three different microorganisms (that form a three-level marine microbial food web) performed efficient and rapid exploitation of the corresponding resource patch. The phytoplankton responded to nutrient patches, bacteria to phytoplankton patches and bacterivores to prey bacteria patches, meaning that microscale nutrient patchiness may trigger the formation of groups of bacteria and protozoa in the ocean.

In these experiments microfluidics was used to engineer spatially distributed ecological interactions that affect interspecific competition in mixed species ecosystems. Such level of control over relevant ecological factors makes microfluidic techniques ideal for experimental validation of model predictions.

## The role of space in community formation and structure

Ecological communities are multispecies assemblages of interacting populations. Community ecology aims to explore how interactions of populations with each other and their abiotic environment determine the development, distribution and functioning of communities. Most microbes in nature live in communities, which are also relevant for medical and biotechnology applications. Microbial communities are thus intensely studied (Widder et al., 2016) and microfluidic systems have great potential in this area of ecology. Microbial communities may be used as model systems to study the fundamental processes shaping multispecies assemblages in general. Together with microfluidic techniques, they can contribute to the research of community ecology by enabling precise control of the microscale spatial structure and biochemical connectivity within the habitat. Stable and active microbial communities can be constructed on microfluidic chips implementing landscapes with different levels of connectivity.

Droplet-based methods proved to be useful in investigating the effect of community size and composition (Park et al., 2011b; Chang et al., 2015; Cao et al., 2017). Ge and co-workers on the other hand created nanoporous microscale microbial incubators that consisted of wells separated by porous walls (Ge et al., 2013). The device permitted co-culturing of multiple species which were in chemical connection but remained physically isolated. The interaction of populations in neighboring chambers was demonstrated by co-culturing quorum sensing pairs (sender and receiver strains). In a similar fashion, a device with three separated wells with communication channels in between can be constructed (Kim et al., 2008). A community of three species of soil bacteria was investigated in this device. The bacteria were incapable of living in well-mixed batch culture conditions, and individual species were also unsuccessful in colonizing the microfabricated device alone. However, under nutrient-limited conditions the mixed community was able to survive and stabilize in the device utilizing syntrophic interactions between community members. The spatial structure of the device was crucial in this stabilization: a few hundred micron separation distance between populations proved to be optimal.

Traditional microfabrication techniques are mostly limited to 2D patterns, complex 3D structures are difficult to achieve. Laser-based micro-3D printing had been successfully applied to create various 3D bacterial arrangements in gelatin (Connell et al., 2013). For example in a nested structure, *Staphylococcus aureus* was protected against ampicillin antibiotic treatments when surrounded with a shell composed of *Pseudomonas aeruginosa*. Similarly, Kim et al. created a core-shell structure of two bacteria in order to remove a mixture of pollutants (Kim et al., 2011). They put together *Sphingobium chlorophenolicum*, a pentachlorophenol degrader, and *Ralstonia metallidurans*, a mercuric ion reducer. They found that the community was able to degrade pentachlorophenol in the presence of the heavy metal only in a spatial structure when *S. chlophenolicum* was protected in the core.

Biofilms are surface-associated structured microbial communities where cell-cell interactions and the microenvironment play a defining role, and therefore microfluidics brought new tools to study these structures in a controlled manner (Kim et al., 2012b; Chang et al., 2015; Yawata et al., 2016). The impact of physiologically relevant factors, such as shear stress, oxygen level, porosity, topology and other constraints can be explored by novel methods (Skolimowski et al., 2010; Park et al., 2011a; Drescher et al., 2013; Hassanpourfard et al., 2014), which are opening new avenues of research, helping us to explore questions that are impossible to address in macroscopic settings. The assembly of dense surface-associated bacterial communities is often a physiological response to external stress. By forming biofilms, bacterial populations can better tolerate and survive in hostile environments (Kim et al., 2010b). Understanding how different biochemical signals influence the organization and structure of biofilms may help us in fighting against bacterial infections. High-throughput microfluidic devices enable screening of compounds that promote or inhibit biofilm formation of pathogenic bacteria under flow or batch conditions (Kim et al., 2012a).

Markov and co-workers demonstrated that liquid flow and inoculation density plays a key role in the formation of bacterial flocks and biofilms in structured microhabitats (Markov et al., 2010). Kim et al. investigated host-pathogen interactions by developing a device for co-culturing epithelial cells and bacteria in a manner that mimics the gastrointestinal tract microenvironment (Kim et al., 2010a). Using this system they reported the importance of signaling molecules (indol) produced by commensal *E. coli* bacteria in the pathogenic EHEC strain colonization.

Oliveira and his colleagues used microfluidic flow cells to study biofilm formation by co-culturing different natural isolates of *P. aeruginosa* strains (Oliveira et al., 2015). They have shown that ecological competition between these strains promotes biofilm formation. Moreover, pyocins, narrow-spectrum antibiotics produced by *P. aeruginosa*, at low concentrations enhanced cell density and volume of these biofilms. Lambert et al. studied biofilm formation of *E. coli* strains in microhabitat arrays (Lambert et al., 2014a). They have shown that signaling mechanisms are fundamental for explaining the behavior and biofilm formation of bacteria in constrained environments. In competition experiments using mixed cultures, they observed spatial segregation of *E. coli* strains in response to starvation stress. Coyte and co-workers used a microfluidic model system consisting of porous environments in order to investigate how hydrodynamics affects the competition between various bacterial growth strategies (Coyte et al., 2017). They used experiments and modeling as well as game theory to show that fast and slow flow conditions favor fast- and slow-growing biofilms, respectively. In other words, surprisingly, in some conditions slower growth grants a competitive advantage. Similar studies in porous media (Nadell et al., 2017) explored the delicate interplay between hydrodynamics, biofilm growth and competition, and found that expanding biofilms sometimes clogged the fluid flow and this allowed the coexistence of sessile and planktonic cells.

These works demonstrate the potential of microfluidic technologies in community ecology. Physical and biological structure and connectivity within the habitat, as well as other aspects of the microenvironment may be readily manipulated. These capabilities give unique opportunities to study community formation and stability in space and time.

## Spatial structure and heterogeneity in evolution

One advantage of experimenting with microfluidic microbial ecosystems, as opposed to field experiments involving higher organisms, is that evolutionarily relevant timescales spanning numerous generations are readily achievable. Although there are only a handful of recent publications on this topic, these works clearly show the potential of the technology to address various questions in evolutionary ecology. The formation of high cell density flocks of swimming bacteria as the possible first step toward antibiotic resistance had been suggested (Hol et al., 2016a), based on evidence from microfluidic studies performed on *E. coli* in antibiotic gradients. The pioneering work by Zhang and his colleagues demonstrated that microfluidic techniques can be used to create complex environments to study microbial evolution of antibiotic resistance (Zhang et al., 2011, 2014). They used a network of microscopic chambers with a heterogeneous antibiotic distribution. The expansion of the population in this structure, as well as the emergence of resistant subpopulations was tracked during the experiments. Remarkably, resistant mutants emerged within a few hours after inoculating the microfluidic chip. Several key mutations were identified in the evolved populations, and these results imply that the spatial complexity of the habitat has a deep impact on the spatiotemporal characteristics of evolutionary processes.

## Conclusions

Artificial microfluidic microbial ecosystems have opened the way toward ecology experiments with unprecedented control over environmental factors and long timescales relative to generation times. These integrated, microfluidic systems hosting cell ecosystems on-chip have proved to be useful not only in exploring the microbial world, but also addressing more general questions in ecology from population dynamics to community formation and function to competition and evolution. The capacity of precise tracking of individuals (cells) and populations, accurately defining the spatial structure of the habitat and dynamically control the environmental parameters are indispensable and lay tremendous power in the hands of the experimenter. Although basic ecosystems have been studied in microfluidic chips, standardization (of microfabricated structures, protocols, etc.) and integration with other analytical techniques (cell-isolation, DNA extractions, sequencing, etc.) are the obvious next steps to expand the spectrum of applications and make the technology mainstream.

Besides serving as a substitute for field experiments, microfluidics can actually help to bridge the distance between laboratory research and field studies, especially in microbiology: new methods are emerging that help us to bring previously unculturable natural strains into the lab (Nichols et al., 2010), or build artificial systems to mimic natural habitats such as soil (Stanley et al., 2016; Aleklett et al., 2017) and the gut microbiome (Shah et al., 2016). New microfluidic devices are being deployed in field experiments as sensors (Nightingale et al., 2015; Grand et al., 2017), sample collection tools (Bouchillon et al., 2014) *in situ* assays (Lambert et al., 2017) or used for analyzing collected samples (Chandler et al., 2013). We expect new applications, novel ways of using chips and new microfluidic devices to appear in the near future. Further progress in the field can make microfluidics a mainstream technology in experimental (microbial) ecology.

## Author contributions

All authors listed have made a substantial, direct and intellectual contribution to the work, and approved it for publication.

### Conflict of interest statement

The authors declare that the research was conducted in the absence of any commercial or financial relationships that could be construed as a potential conflict of interest. The reviewer RP-M and handling Editor declared their shared affiliation.
